# Fibrinolytic therapy use for ST-segment elevation myocardial infarction and long-term outcomes in China: 2-year results from the China Acute Myocardial Infarction Registry

**DOI:** 10.1186/s12872-023-03105-1

**Published:** 2023-02-22

**Authors:** Chao Wu, Ling Li, Shuqing Wang, Jianping Zeng, Jingang Yang, Haiyan Xu, Yanyan Zhao, Yang Wang, Wei Li, Chen Jin, Xiaojin Gao, Yuejin Yang, Shubin Qiao

**Affiliations:** 1 Coronary Heart Disease Center, Department of Cardiology, State Key Laboratory of Cardiovascular Disease, National Center for Cardiovascular Diseases, Chinese Academy of Medical Sciences and Peking Union Medical College, Fuwai Hospital, No.167 BeiLiShi Rd, Xicheng District, Beijing, 100037 People’s Republic of China; 2 Medical Research & Biometrics Center, State Key Laboratory of Cardiovascular Disease, National Center for Cardiovascular Diseases, Chinese Academy of Medical Sciences and Peking Union Medical College, Fuwai Hospital, Beijing, People’s Republic of China; 3 Internal Medicine‑Cardiovascular Department, The First Hospital of Qiqihar, Affiliated Qiqihar Hospital, Southern Medical University, Qiqihar, Heilongjiang Province People’s Republic of China; 4 Department of Cardiology, Xiangtan Central Hospital, Xiangtan, Hunan Province People’s Republic of China

**Keywords:** ST-segment elevation myocardial infarction, Fibrinolysis, Outcomes

## Abstract

**Background:**

Data on fibrinolytic therapy use for ST-segment elevation myocardial infarction (STEMI) and long-term clinical outcomes in developing countries are limited. We aimed to investigate the management and 2-year mortality of fibrinolytic-treated patients in China.

**Methods:**

A total of 19,112 patients with STEMI from 108 hospitals participated in the China Acute Myocardial Infarction registry between January 2013 and September 2014. We investigated the 2-year all-cause mortality among patients treated with fibrinolysis. Non-invasive clinical indexes were used to diagnose successful fibrinolysis or not.

**Results:**

Only 1823 patients (9.5%) enrolled in the registry underwent fibrinolysis and 679 (37.2%) could be treated within 3 h after symptom onset. The overall use of rescue percutaneous coronary intervention was 8.9%. Successful fibrinolysis, which could be achieved in 1428 patients (78.3%), was related to types of fibrinolytic agents, symptom to needle time, infarction site, and Killip class. Follow-up data were available for 1745 patients (95.7%). After multivariate adjustment, successful fibrinolysis was strongly associated with a decreased risk of death compared with failed fibrinolysis at 2 years (8.5% vs. 29.0%, hazard ratio: 0.27, 95% confidence interval: 0.20–0.35).

**Conclusion:**

Within a minority of STEMI patients in the CAMI registry underwent fibrinolysis, most of them could achieve successful clinical reperfusion, presenting a much benign 2-year survival outcome than those with failed fibrinolysis. Quality improvement initiatives focusing on fibrinolysis are warranted to achieve its promise fully.

*Trial registration*: URL: https//www.clinicaltrials.gov. Unique identifier: NCT01874691. Registered 11/06/2013.

**Supplementary Information:**

The online version contains supplementary material available at 10.1186/s12872-023-03105-1.

## Introduction

Although primary percutaneous coronary intervention (PCI) has replaced fibrinolysis as the preferred reperfusion strategy in the current management of ST-segment elevation myocardial infarction (STEMI) [1, 2], the latter is still the mainstay in regions with challenging geography and limited healthcare provision, particularly in developing countries [3–9]. However, compared with detailed reports of fibrinolysis utilization and clinical outcomes in treated patients in developed countries [10–12], the management and prognosis of fibrinolytic-treated patients in developing countries have been previously investigated in either randomized trials, which might not be representative of real world clinical practice [13, 14], or in registry studies that either restricted to examining modest-sized cohorts [15], a single-center design [15, 16], or relatively short-term follow-up [6, 16, 17]. Furthermore, most of them were performed in the era preceding the use of current adjunctive antiplatelet therapy [13–17]. Considering the substantial discrepancies in the total STEMI burden have persisted between different regions over the past ten years, appearing to be higher in developing countries [18], further improvements in the survival outcome of fibrinolytic-treated patients could be significantly important to enhancing STEMI health worldwide.

Utilizing data derived from the China Acute Myocardial Infarction (CAMI) registry, we sought to evaluate the management and long-term mortality of a large cohort of STEMI patients treated with fibrinolytic therapy in this largest developing country.

## Methods

### Overview of the CAMI registry design and study population

The design of the CAMI registry has been previously reported in detail [19, 20]. Briefly, a total of 108 hospitals in 27 provinces and 4 municipalities in Mainland China participated (Additional file 1), enrolling 26,648 AMI patients between January 2013 and September 2014. These hospitals included 31 province-level hospitals (all university-affiliated academic hospitals located in capital city of each province), 45 prefecture-level hospitals (in medium-sized cities), and 32 county-level hospitals (in the smallest cities usually with surrounding rural areas), with broad coverage of geographical regions. There were obvious differences among provincial-level, prefectural-level and country-level hospitals for the annual admission of AMI which was 400, 232, and 80 cases, fibrinolysis proportion which was 88.2%, 100% and 91.4%, PCI proportion which was 100%, 89.1% and 37.1%, and primary PCI proportion which was 100%, 84.8% and 31.4%.

Patients with STEMI treated with fibrinolytic therapy were included in our study. STEMI was diagnosed following the Third Universal Definition for Myocardial Infarction, including types 1, 2, 3, 4b, and 4c [21]. Type 4a and type 5 AMIs were not eligible for the CAMI registry.

### Data collection and management

All information in the CAMI registry was collected using a standardized set of variables and predefined, standard, unified definitions, systematic data entry and transmission procedures, and rigorous data quality control. Data were collected, validated, and submitted through a secure, web-based electronic data capture system. Enrollment, data collection, and follow-up were all performed by trained physicians at each participating site in a real-time manner, to ensure data accuracy and reliability. Senior cardiologists were responsible for the data quality control. Periodic database checking was undertaken. Hospital sites underwent random on-site audits for the accuracy of diagnosis and variables based on medical records.

This study was approved by the institutional review board central committee at Fuwai Hospital. Written informed consent was obtained from eligible patients, and the study protocol conformed to the ethical guidelines of the 1975 Declaration of Helsinki. All methods were carried out in accordance with relevant guidelines and regulations.

### Variables in care and outcomes

The use and dosage of fibrinolytic agents were based on the Chinese guideline of STEMI management but finally at the discretion of the physicians [22]. Inappropriate dosage included overdosing or underdosing. The successful clinical reperfusion after fibrinolysis was assessed according to non-invasive indexes and was determined if any two of the following four items (3 + 4 excluded) could be achieved: (1) ST-segment resolution ≥ 50% within 60–90 min of receiving fibrinolytic therapy; (2) the time to peak and cardiac troponin concentration is advanced to ≤ 12 h of symptom onset and the time to creatine kinase-MB concentration is advanced to ≤ 14 h of symptom onset; (3) significant relief of chest pain within 2 h of fibrinolytic therapy; (4) presence of reperfusion arrhythmia within 2 to 3 h of fibrinolytic therapy, including accelerated idioventricular rhythm, sudden improvement or disappearance of atrioventricular block or bundle branch block, and transient sinus bradycardia or sino-auricular block with or without hypotension among patients with inferior wall MI [22].

The primary outcome was 2-year all-cause death. The secondary outcome was all-cause death, reinfarction, stroke, a drop in hemoglobin ≥ 5 g/dL, and intracranial hemorrhage during hospitalization.

### Statistical analysis

Continuous variables were expressed as median and interquartile range (IQR) and were compared using analysis of variance or the Kruskal–Wallis test. Categorical variables were expressed as numbers and percentages and were compared using the Pearsonχ^2^ test or Fisher’s exact test. Survival curves were constructed using the Kaplan–Meier method and compared using the log-rank test. Multivariable Cox proportional-hazards models were used to assess the risk of successful fibrinolysis to failed fibrinolysis for the primary outcomes, expressed as hazard ratios (HR) and its 95% confidence interval (CI). The adjusted variables included age (≤ 60 vs. > 60 years), sex, hypertension, diabetes, current smoking, Killip class (≥ II vs. I), symptom to needle time (< 3 vs. ≥ 3 h), anterior infarction, use of fibrin-specific agents, and receiving rescue PCI. Multivariate logistic regression analysis was performed to determine the independent predictors of successful fibrinolysis using the above-adjusted variables except the use of rescue PCI, expressing as odds ratios (OR) and its 95% CI.

All tests of significance were two-tailed, and P < 0.05 was considered statistically significant. The analyses were performed using SAS 9.4 (SAS Institute, Cary, NC, USA).

## Results

**Fig. 1:**
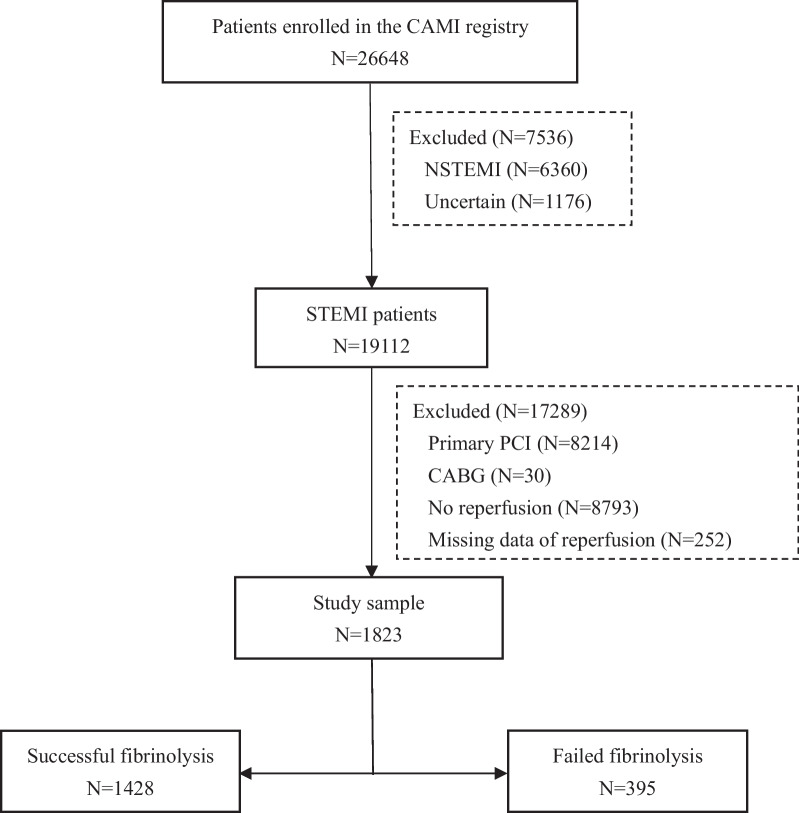
Study flow. CAMI: China Acute Myocardial Infarction; NSTEMI: non-ST-segment elevation myocardial infarction; STEMI: ST-segment elevation myocardial infarction; PCI: percutaneous coronary intervention; CABG: coronary artery bypass grafting

Of 19,112 patients with STEMI registered in the CAMI registry from January 1, 2013, through September 30, 2014, we excluded 8214 with primary PCI, 30 with coronary artery bypass grafting, 8793 without reperfusion therapy, and 252 without available data on reperfusion therapy. Thus, a total of 1823 patients (9.5%) treated with fibrinolysis were included in the study (Fig. 1).

**Fig. 2:**
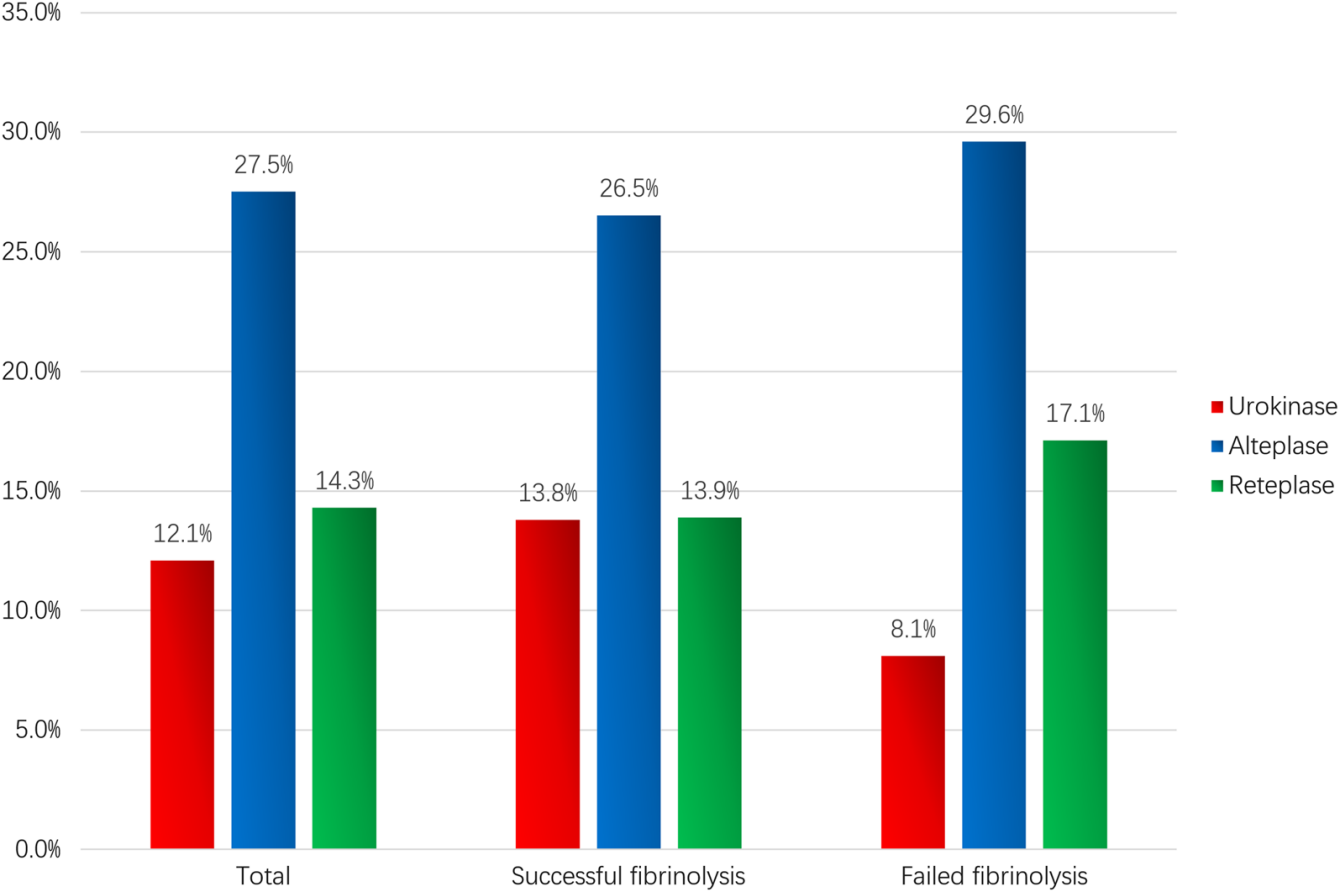
Types and inappropriate dosage of fibrinolytic agents

The baseline characteristics are shown in Table 1. Most of the study sample were admitted in prefectural-level (n = 992, 54.4%) and county-level hospitals (n = 664, 36.4%). The overall median age of the cohort was 60 y and 78.4% were male. The median symptom to needle time was 220 min and 672 patients (37.2%) could achieve reperfusion within 3 h after symptom onset. More than 90% of patients received aspirin and clopidogrel during hospitalization. Non-fibrin-specific agents (including streptokinase and urokinase) and fibrin-specific agents (including alteplase and reteplase) were used for 38.3% and 61.7% of patients respectively. Administration of inappropriate dosage of fibrinolytic agents was varied: 12.1% for urokinase, 27.5% for alteplase, and 14.3% for reteplase (Fig. 2). Dosage information of streptokinase was unavailable.

**Table 1:** Baseline Characteristics

	Total (n = 1823)	a) Successful fibrinolysis (n = 1428)	b) Failed fibrinolysis (n = 395)	P value (a vs. b)
Age (y)	60.3(51.4,67.8)	60.0(51.0,67.3)	61.7(54.9,68.8)	0.010
≤ 60 y	874(47.9%)	717(50.2%)	157(39.7%)	< 0.001
Male	1430(78.4%)	1145(80.2%)	285(72.2%)	< 0.001
Hospital level				0.441
Provincial level	167 (9.2%)	130 (9.1%)	37 (9.4%)	
Prefectural level	992 (54.4%)	788 (55.2%)	204 (51.6%)	
County level	664 (36.4%)	510 (35.7%)	154 (39.0%)	
Diabetes	250 (13.7%)	190 (13.3%)	60 (15.2%)	0.340
Hypertension	831 (45.6%)	642 (45.0%)	189 (47.8%)	0.308
Current smoking	934 (51.2%)	758 (53.1%)	176 (44.6%)	0.003
Prior MI Prior stroke	120 (6.6%) 142 (7.8%)	100 (7.0%) 108 (7.6%)	20 (5.1%) 34 (8.6%)	0.157 0.498
Killip class ≥ II	351 (19.3%)	241 (16.9%)	110 (27.8%)	< 0.001
Medication during hospitalization				
Aspirin	1790 (98.2%)	1406 (98.5%)	384(97.2%)	0.119
Clopidogrel	1771 (97.1%)	1394 (97.6%)	377(95.4%)	0.030
Unfractionated heparin	175 (9.6%)	126 (8.8%)	49(12.4%)	0.038
Low molecular weight heparin	1628 (89.3%)	1296 (90.8%)	332 (84.1%)	< 0.001
Beta-blocker	1186(65.1%)	947(66.3%)	239 (60.5%)	0.033
Statin	1673 (91.7%)	1308(91.4%)	365 (92.4%)	0.602
Anterior infarction	887 (48.7%)	650(45.5%)	237 (60.0%)	< 0.001
Fibrinolytic agents	(n = 1797)	(n = 1413)	(n = 384)	< 0.001
streptokinase	16 (0.9%)	12 (0.8%)	4 (1.0%)	
urokinase	672 (37.4%)	463 (32.8%)	209 (54.5%)	
alteplase	325 (18.1%)	268 (19.0%)	57 (14.8%)	
reteplase	784 (43.6%)	670 (47.4%)	114 (29.7%)	
Symptom to needle time (min) < 3 h	220 (122,305) 679 (37.2%)	212 (112,306) 563 (39.4%)	246 (186,366) 116 (29.4%)	0.318 < 0.001
Rescue PCI	162 (8.9)	82 (5.7%)	80 (20.3%)	< 0.001

Data are median (interquartile range) and n/total n (%)

*MI* myocardial infarction; *PCI* percutaneous coronary intervention

1428 patients (78.3%) achieved successful fibrinolysis, who were relatively younger (median age [y]: 60.0 vs. 61.7, P = 0.010) and more likely to be male (80.2% vs. 72.2%, P < 0.001). Furthermore, Killip class ≥ II (16.9% vs. 27.8%) and anterior infarction (45.5% vs. 60.0%, P < 0.001) were less common in this group. Patients with successful fibrinolysis were more likely to use fibrin-specific agents (66.4%), while 55.5% of patients with failed fibrinolysis received non-fibrin-specific agents. After fibrinolysis, rescue PCI was infrequently performed in both groups: 5.7% for successful fibrinolysis versus 20.3% for failed fibrinolysis (P < 0.001) (Table 1).

**Fig. 3:**
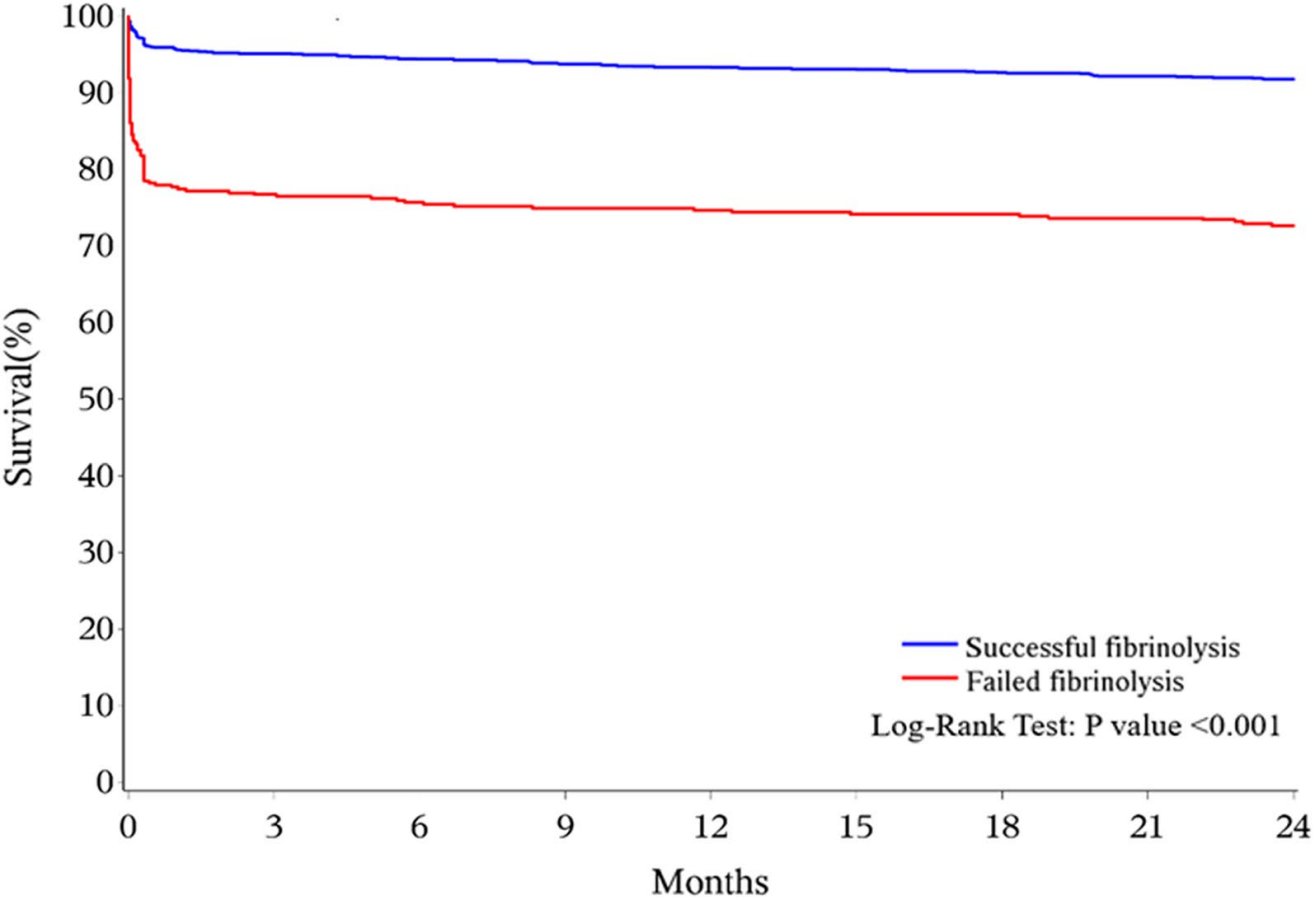
Kaplan–Meier graphs for 2-year all-cause death

Clinical outcomes are detailed in Table 2. In-hospital all-cause death was less common in patients with successful fibrinolysis (3.7% vs. 21.5%, P < 0.001). Other adverse events during hospitalization were rare and there were no significant differences between two groups. Follow-up data were available for 1745 patients (95.7%) and the overall 2-year mortality was 12.9%. Deaths occurred within 1-month comprised a considerable proportion of the total death number in either successful or failed fibrinolysis group during the follow-up: 53% for the former and 81% for the latter (Fig. 3). After multivariate adjustment, successful fibrinolysis was strongly associated with a decreased risk of death at 2 years compared with failed fibrinolysis (8.5% vs. 29.0%, HR: 0.27, 95% CI 0.20–0.35) (Table 3). Other independent predictors of death were age ≤ 60y (HR: 0.46, 95% CI 0.33–0.64), Killip class ≥ II (HR: 1.82, 95% CI 1.38–2.41), and the use of rescue PCI (HR: 0.23, 95% CI 0.16–0.48).

**Table 2:** Clinical outcomes

	Total (n = 1823)	a) Successful fibrinolysis (n = 1428)	b)Failed fibrinolysis (n = 395)	P value (a vs. b)
*Unadjusted in-hospital event rates*				
All-cause death	138/1823(7.6%)	53/1428(3.7%)	85/395(21.5%)	< 0.001
Reinfarction	22/1820 (1.2%)	15/1427 (1.1%)	7/393 (1.8%)	0.293
Stroke	18/1820 (1.0%)	14/1427 (1.0%)	4/393 (1.0%)	1.000
Hemoglobin drop ≥ 5 g/dL	6/1823 (0.3%)	4/1428 (0.3%)	2/395 (0.5%)	0.616
Intracranial hemorrhage	9/1823 (0.5%)	8/1428 (0.6%)	1/395 (0.3%)	0.693
*Unadjusted mortality during follow-up*				
30 days	150/1805 (8.3%)	62/1416 (4.4%)	88/389 (22.6%)	< 0.001
6 months	176/1790 (9.8%)	80/1405 (5.7%)	96/385 (24.9%)	< 0.001
1 year	195/1779 (11.0%)	95/1397 (6.8%)	100/382 (26.2%)	< 0.001
2 years	225/1745 (12.9%)	117/1372 (8.5%)	108/373 (29.0%	< 0.001

Data are n/total n (%)

**Table 3:** Independent predictors of two-year all-cause death

	HR (95% CI)	P value
Successful fibrinolysis	0.27 (0.20–0.35)	< 0.001
Age ≤ 60 y	0.46 (0.33–0.64)	< 0.001
Male	0.75 (0.55–1.02)	0.066
Hypertension	1.14 (0.87–1.49)	0.349
Diabetes	1.21 (0.86–1.70)	0.269
Current smoking	0.92 (0.67–1.25)	0.583
Killip class ≥ II	1.82 (1.38–2.41)	< 0.001
Symptom to needle time < 3 h	0.77 (0.57–1.04)	0.083
Anterior infarction	1.24 (0.95–1.62)	0.113
Fibrin-specific agents	1.29 (0.98–1.69)	0.073
Rescue PCI	0.23 (0.16–0.48)	< 0.001

*HR* hazard ratio; *CI* confidence interval; *PCI* percutaneous coronary intervention

In the logistic regression model shown in Table 4, symptom to needle time < 3 h (OR: 1.40, 95% CI 1.09–1.80) and the use of fibrin-specific agents (OR: 2.47, 95% CI 1.96–3.12) were independent predictors of successful fibrinolysis, whereas Killip class ≥ II (OR: 0.58, 95% CI 0.44–0.76) and anterior infarction (OR: 0.56, 95% CI 0.44–0.71) were strongly associated with an increased risk of failed fibrinolysis.

**Table 4:** Independent predictors of successful fibrinolysis

	OR (95% CI)	P value
Age ≤ 60 y	1.22 (0.95–1.57)	0.111
Male	1.34 (1.00–1.79)	0.052
Hypertension	0.99 (0.78–1.26)	0.942
Diabetes	0.91 (0.65–1.27)	0.579
Current smoking	1.06 (0.81–1.37)	0.679
Killip class ≥ II	0.58 (0.44–0.76)	< 0.001
Symptom to needle time < 3 h	1.40 (1.09–1.80)	0.008
Anterior infarction	0.56 (0.44–0.71)	< 0.001
Fibrin-specific agents	2.47 (1.96–3.12)	< 0.001

*OR* odds ratio; *CI* confidence interval

## Discussion

In contrast with prior studies reported in other developing countries, this CAMI registry sub-analysis is the first to address the relation between fibrinolytic therapy and late survival in a large cohort of STEMI patients in contemporary daily practice. We found that less than 10% of STEMI patients enrolled in the registry were treated with fibrinolysis, which was being suboptimally used largely due to the widely use of non-fibrin-specific agents, inappropriate dosage, treatment delay, and the shortfall of necessary cardiovascular intervention*. S*uccessful fibrinolysis, which was a strong and independent predictor of 2-year mortality, could be achieved in nearly 80% of patients. Although failed fibrinolysis was relatively rare, this population experienced more than a 3.4-fold mortality risk at 2 years than those with successful fibrinolysis.

Among STEMI patients enrolled in the CAMI registry, only 1/10 were treated with fibrinolysis, due to the relatively lower annual STEMI admission and less chance to receive reperfusion therapy caused by the longer prehospital delay in prefectural- and county-level hospitals, in which most of the fibrinolysis-treated patients (> 90%) in the present study were admitted in [20, 23]. Nevertheless, fibrinolysis is still the mainstay reperfusion strategy for STEMI in resource-poor settings without advanced technology or access to specialized care, especially in county-level hospitals, the major force in delivering basic public health services in rural China which directly provide inpatient care for nearly half of the national population [23, 24].

Compared with the previous survey of the China Patient-centered Evaluative Assessment of Cardiac Events Retrospective Study of AMI (China PEACE-retrospective AMI study) [17], a vast majority (> 97%) of the present cohort received clopidogrel, and there was a marked increase in the use of fibrin-specific agents (from 3.8 to 61.7%), suggesting subjects in the present study were more likely to achieve coronary patency and at lower risk of coronary reocclusion than those in the prior study. Concomitantly, compared with the report of the Collaborative Research Group on Thrombolysis which used urokinase in all subjects without using a P2Y12 inhibitor, successful clinical reperfusion strongly increased in the present cohort (from 66.5 to 78.3%) [13], associating with 1-year mortality (6.8%) comparable with those treated with PPCI in the large Swedish Register of Information and Knowledge about Swedish Heart Intensive Care Admissions (RIKS-HIA) (7.6%) [10] and the French registry of Acute ST-elevation and non–ST-elevation Myocardial Infarction (FAST-MI) (8.2%) [11].

Such relatively benign survival outcomes observed in patients with successful clinical reperfusion after fibrinolysis supported that clinical judgment criteria of reperfusion were simple, inexpensive, and reliable to stratify patients by the risk of long-term mortality shortly after fibrinolysis in actual practice, and their use should be strongly encouraged in regions with limited catheterization laboratories. Hence, it becomes of significant importance to characterize clinical factors that are associated with successful clinical reperfusion. This issue has been poorly studied in previous studies. In the present study, we found that the use of fibrin-specific agents and symptom to needle time < 3 h were independent predictors of successful fibrinolysis, suggesting that substantial room for improving the long-term prognosis in fibrinolytic-treated patients remains at a national level: (1) Although there has been evidence of substantial improvement in the use of fibrin-specific agents, urokinase, which was inferior with regard to the patency of the infarct-related artery among the fibrinolytic therapy agents available [14, 25], was still used in more than 1/3 of treated patients. (2) The symptom to needle time < 3 h, which is recommended by the Chinese guideline, was only achieved in 37.2% of patients. And the median symptom to needle time was much longer than in other national databases. Such suboptimal fibrinolytic regimens and treatment delays have been also reported by other developing countries with even worse results (i.e., predominantly streptokinase use and longer total ischemic time) [3–5, 7–9], in sharp contrast with North America and Europe [10–12].

Notably, the advanced Killip class and anterior infarction were associated with a higher risk of failed fibrinolysis. Although the mechanisms underlying this phenomenon were not addressed by the present (or any prior) study, it is possible that those with heart failure at presentation or anterior infarction may suffer from a larger infarction, which may be associated with more severe damage of microcirculation, and thus impaired perfusion [26].

Interestingly, we found deaths within 30 days comprised a considerable part of the total death number at 2 years. Such findings highlighted the need for a quality improvement initiative with a clear focus on this high risk period of death (within 30 days, especially during hospitalization). Furthermore, the proportion of short-term deaths varied from the successful fibrinolysis group (53%) to the failed fibrinolysis group (81%), suggesting the difference in mortality risk existed between these two groups during the long-term follow-up.

The use of fibrinolytic drugs alone without performing necessary adjunctive revascularization appears to be another important issue. In the present study, rescue PCI was performed in 8.9% of the entire cohort, much less than the results reported by the Saudi AMI Registry Program (38%) [27], the French FAST-MI registry (58%) [11], and the US National Cardiovascular Data Registry (41.5%) [12]. Of note, only ~ 20% of patients with failed fibrinolysis underwent rescue PCI in the present study, which likely accounts, at least in part, for the poor survival outcomes in this group, given the short- and long-term prognostic benefits associated with rescue PCI for failed fibrinolysis has been proved in either the clinical trial or registry study [28, 29]. This issue may be ascribed to the following reasons.: (1) a vast majority (> 90%) were admitted in prefectural-level and county-level hospitals, with a relatively poorer capacity for cardiovascular intervention of AMI than provincial-level hospitals, particularly regarding primary PCI; (2) Before the healthcare reform act for standardizing the use and price of stents enforcing in 2020, the total PCI-related cost during hospitalization was much higher than fibrinolysis [30]. Such out-of-pocket costs might keep a number of patients from taking further interventional therapy even after failed fibrinolysis.

Considering that fibrinolytic therapy is still being largely used in rural areas or other regions with limited medical conditions, geographical location, and techniques, a group of coordinated regional management protocols have been investigated in a real-world setting for developing STEMI systems of care through greater use of reperfusion strategy, associating with improving survival outcomes [31–33]. Accordingly, strategies targeting improving the current suboptimal treatment patterns in China and other developing countries which are facing the similar situation, should focus on improving the use of fibrin-specific agents and post-treatment cardiovascular intervention, as well as popularizing the public knowledge of STEMI, upgrading the transportation facilities, and extensively training of physicians and prehospital staff, for decreasing the treatment delay [34–36]. Deeper cooperation among different-level healthcare institutes might be important for utilizing the pharmacoinvasive strategy.

Several limitations should be mentioned in this study. First, the registry data regarding to the management of fibrinolysis-treated patients were collected nearly 10 years ago. Further studies with greater timeliness are required. However, given that fibrinolytic therapy is still an irreplaceable reperfusion therapy in the current management of STEMI, the relevant findings of this study are still of great significance for identifying high risk patients and improving the prognosis in this population. Second, results of the anatomic infarct-related artery patency after fibrinolytic therapy were lack in this study, so the relationship between the clinical and angiographic signs of reperfusion could not be evaluated. Third, the analysis of electrocardiogram data was performed by clinical physicians but not in a core laboratory, which might be less objective and accurate. However, we established a definite protocol to read the electrocardiograms and to assess ST-segment resolution that was common to all participating centers. The absence of the causes of death for those who died during the long-term follow-up was another notable limitation. Finally, as an observational study, although several statistical adjustments were performed, we could not exclude the presence of unmeasured selection bias, given most of the STEMI patients in this registry were excluded.

## Conclusion

Our study fills an important gap in the literature as there have been no prior large real-world reports on the long-term prognosis after fibrinolytic therapy among STEMI patients in developing countries. In this nationwide survey of patients with STEMI treated with fibrinolytic therapy, successful clinical reperfusion could be achieved in a majority of this population, yielding a significantly lower risk of 2-year all-cause mortality compared with failed fibrinolysis. Significant underuse and suboptimal administration of fibrinolytic therapy for STEMI, which may undermine the potential benefit of this therapy for patients treated in facilities without, still need to be concerned.

## Supplementary Information

**Additional file 1**.** eAppendix 1**. Full List of Hospitals in the CAMI Registry.

## Data Availability

The datasets used and/or analyzed during the current study are available from the corresponding author upon reasonable request.
