# Supplementary material for: Fibrinolytic therapy use for ST-segment elevation myocardial infarction and long-term outcomes in China: 2-year results from the China Acute Myocardial Infarction Registry
Source: BMC Cardiovasc Disord. 2023 Feb 22;23:103. doi: 10.1186/s12872-023-03105-1 (PMC9948459; doi:10.1186/s12872-023-03105-1)
Supplement: Supplementary file 1 — Additional file 1. eAppendix 1. Full List of Hospitals in the CAMI Registry. [file 12872_2023_3105_MOESM1_ESM.pdf]

eAppendix 1. Full List of Hospitals in the China AMI Registry

Hospital	Province/Municipality	City	PI
Fuwai Hospital	Beijing	Beijing	Yuan Wu
Beijing Friendship Hospital	Beijing	Beijing	Hongwei Li
Beijing Tongren Hospital	Beijing	Beijing	Changlin Lu
Beijing Daxing Hospital	Beijing	Daxing	Shujun Cao
Beijing Mentougou Hospital	Beijing	Mentougou	Dezhao Wang
Beijing Pinggu Hospital	Beijing	Pinggu	Guanglin Wei
Beijing Yanqing Hospital	Beijing	Yanqing	Jianbing Wang
Shanghai Jiaotong University Ruijin Hospital	Shanghai	Shanghai	Ruiyan Zhang
Shanghai 10th Hospital	Shanghai	Shanghai	Yawei Xu
Shanghai Fengxian Hospital	Shanghai	Fengxian	Zengyong Qiao
Tianjin Medical School General Hospital	Tianjin	Tianjin	Zheng Wan
Tianjin Baodi Hospital	Tianjin	Baodi	Yanjun Cao
Chongqing Medical School 2st Hospital	Chongqing	Chongqing	Yaohui Yin
Haerbin Medical School 1st Affiliated Hospital	Heilongjiang	Harbin	Weiming Li
Qiqihaer 1st Hospital	Heilongjiang	Qiqihar	Shuqing Wang
Tailai Hospital	Heilongjiang	Tailai	Gang Ma
Shuihua 1st Hospital	Heilongjiang	Shuihua	Yongchen Cai
Jilin University 1st Hospital	Jilin	Changchun	Yang Zheng
Tonghua Central Hospital	Jilin	Tonghua	Xuxia Zhang
Huinan County Hospital	Jilin	Huinan	Hongyan Guo
Shenyang Northern Hospital	Liaoning	Shenyang	Xiaozeng Wang
Fushun Central Hospital	Liaoning	Fushun	Ling Sun
Xiuyan County Hospital	Liaoning	Xiuyan	Jianhua Wu
Neimonggu Medical College 1st Affiliated Hospital	Inner Mongolia	Hohhot	Fengying Chen
Chifeng Hospital	Inner Mongolia	Chifeng	Ronghai Man
Aohan Hospital	Inner Mongolia	Aohan	Yanjie Li
Hebei Medcial School 2rd Affiliated Hospital	Hebei	Shijiazhuang	Xianghua Fu
Qinhuangdao 1st Hospital	Hebei	Qinhuangdao	Qingshen Wang
Qinhuangdao 2rd Hospital	Hebei	Changli	Liying Zhang
North-China Oil-administration General Hospital	Hebei	Renqiu	Xiaoli Gao
Changzhou Hospital	Hebei	Changzhou	Yali Hu
Hengshui Hardison Hospital	Hebei	Hengshui	Qun Zheng
Shanxi Cardiovascular Hospital	Shanxi	Taiyuan	Bao Li
Changzhi Hospital	Shanxi	Changzhi	Yuping zhang
Tunliu Hospital	Shanxi	Tunliu	Yaohong Dong
Henan Provincial Hospital	Henan	Zhengzhou	Chuanyu Gao
Linzhou Hospital	Henan	Linzhou	Zhoushun Qin
Changyuan Hospital	Henan	Changyuan	Guorui Hou
Xinxiang Central Hospital	Henan	Xinxiang	Lingling Liu
Yanjin Hospital	Henan	Yanjin	Shifeng Ren
Ye County hospital	Henan	Ye County	Dezhou wang
Pindingshan 2rd Hospital	Henan	Pindingshan	Xianting Luan
Anyang Prefecture Hospital	Henan	Anyang	Hui Liu
Puyang People’s Hospital	Henan	Puyang	Liping Ma

© 2020 Xu H et al. *JAMA Network Open.*

Hospital	Province/Municipality	City	PI
Xihua Hospital	Henan	Xihua	Chuntong Wang
Xi'an Jiaotong University 1st Hospital	Shan'xi	Xi'an	Zuyi Yuan
Weinan Central Hospital	Shan'xi	Weinan	Junnong Li
Jiuquan Hospital	Gansu	Jiuquan	Yaofeng Yuan
Jinta Hospital	Gansu	Jinta	Huide Liu
Ningxia Medical College General Hospital	Ningxia	Yinchuan	Shaobin jia
Wuzhong Hospital	Ningxia	Wuzhong	Xianghong Luo
Qinghai University Affiliated Hospital	Qinghai	Xining	Yin Liu
Qinhai Cardiovascular Hospital	Qinghai	Xining	Pinfa Liu
Xining 1st Hospital	Qinghai	Xining	Xianning Zhao
Hainan Prefectural Hospital of Qinghai	Qinghai	Gonghe	Bao Ma
Xinjiang Medical College 1st Affiliated Hospital	Xinjiang	Urumchi	Yitong Ma
Changji Hospital	Xinjiang	Changji	Mao Wang
Fukang Hospital	Xinjiang	Fukang	Shiming Gao
Urumchi Friendship Hospital	Xinjiang	Urumchi	Hang Lu
Shandong Provincial Hospital	Shandong	Jinan	Lianqun Cui
Taian Central Hospital	Shandong	Taian	Huanyi Zhang
Xintai Hospital	Shandong	Xintai	Hongyan Zhang
Nanjing University Gulou Hospital	Jiangsu	Nanjin	Biao Xu
Jiangsu North Hospital	Jiangsu	Yangzhou	Shenghu He
Xuzhou 1st Central Hospital	Jiangsu	Xuzhou	Qiang Fu
Jiangyan Hospital	Jiangsu	Jiangyan	Shihai Shen
Anhui Provincial Hospital	Anhui	Hefei	Likun Ma
Fuyang Hospital	Anhui	Fuyang	Bin Ning
Taihe Hospital	Anhui	Taihe	Jili Fan
Zhejiang University 2rd Affiliated Hospital	Zhejiang	Hangzhou	Yong Sun
Taizhou Enze medical Center	Zhejiang	Taizhou	Lijiang tang
Taizhou Hospital	Zhejiang	Linhai	Danlei Xu
Fujian Medical College Union Hospital	Fujian	Fuzhou	Lianglong Chen
Xiamen Heart Center	Fujian	Xiamen	Yan Wang
Fuqing Hospital	Fujian	Fuqing	Ping chen
Longyan 1st Hospital	Fujian	Longyan	Kaihong Chen
Wuhan Tongji Hospital	Hubei	Wuhan	Daowen wang
Jinzhou 1st Hospital	Hubei	Jinzhou	Shuixian peng
Tianmen 1st Hospital	Hubei	Tianmen	Shuping Wan
Gong'an Hospital	Hubei	Gongan	Laxi Zhang
Central South University Xiangya 2ndHospital	Hunan	Changsha	Shenhua Zhou
Xiangtan Central Hospital	Hunan	Xiangtan	Jianping Zeng
Xiangxiang Hospital	Hunan	Xiangxiang	Chonglun Zhou
Ya'an Hospital	Sichuan	Ya'an	Haibo zhang
Zigong 1st Hospital	Sichuan	Zigong	Dechao Zhong
Danleng County Hospital	Sichuan	Danleng	Yuquan Xiao
Guangxi Medical College 1st Affiliated Hospital	Guangxi	Nanning	Lang Li
Beihai Hospital	Guangxi	Beihai	Hai Zhu
Hepu Hospital	Guangxi	Hepu	Meisheng Lai
Nanchang Universuty 2ndAffiliated Hospital	Jiangxi	Nanchang	Xiaoshu Cheng
**Hospital**	**Province/Municipality**	**City**	**PI**

© 2020 Xu H et al. *JAMA Network Open.*

Pingxiang Hospital	Jiangxi	Pingxiang	Junming Ye
Shangli Hospital	Jiangxi	Shangli	Qishou Liu
Guizhou Cardiovascular Hospital	Guizhou	Guiyang	Tianhe Yang
Zhunyi 1st Hospital	Guizhou	Zhunyi	Zhengqiang Yuan
Honghuagang Hospital	Guizhou	Honghuagan g	Chengyuan Zhao
Pan County Hospital	Guizhou	Pan	Xianwen Jiang
Guangdong Provincial Hospital	Guangdong	Guangzhou	Jiyan Chen
Guangzhou Traditional Chinese Medical College 1st Affiliated Hospital	Guangdong	Guangzhou	Wei Wu
Jiangmen Hospital	Guangdong	Jiangmen	Gaoxing Zhang
Heshan Hospital	Guangdong	Heshan	Haiyuan Mai
Kunming Medical College 1st Affiliated Hospital	Yunnan	Kunming	Tao Guo
Yunnan St. John's Hospital	Yunnan	Kunming	Yi Li
Chuxiong People's Hosptal	Yunnan	Chuxiong	Xiaoming Liu
Yao'an Hospital	Yunnan	Yao'an	Jinlong Xu
Tibet People's Hospital	Tibet	Lahsa	Gesang Luobu
Hainan Provincial Hospital	Hainan	Haikou	Bin Li
Sanya Hospital	Hainan	Sanya	Tiansong Wang
Wenchang Hospital	Hainan	Wenchang	Dong Wang

© 2020 Xu H et al. *JAMA Network Open.*
